# Association between dietary patterns and sarcopenia among community-dwelling older adults in five provinces of China: a cross-sectional study

**DOI:** 10.3389/fpubh.2025.1556033

**Published:** 2025-02-28

**Authors:** Rongchang Pu, Shanshan Jia, Xiaona Zhang, Qingqing Man, Dongmei Yu, Shuya Cai, Pengkun Song, Jian Zhang

**Affiliations:** ^1^Department of Geriatrics and Clinical Nutrition, National Institute for Nutrition and Health, Chinese Center for Diseases Control and Prevention, Beijing, China; ^2^Key Laboratory of Public Nutrition and Health, National Health Commission of the People’s Republic of China, Beijing, China; ^3^Department of Epidemiology Nutrition, National Institute for Nutrition and Health, Chinese Center for Diseases Control and Prevention, Beijing, China

**Keywords:** sarcopenia, dietary patterns, older adults, muscle strength, muscle mass, muscle function

## Abstract

**Background:**

Sarcopenia is prevalent in older adults and not only severely affects their health, but also brings a greater economic burden on the patient’s family as well as society. High-quality diet is one of influencing factors of sarcopenia, particularly important for muscle mass and function. This study aims to examine the dietary patterns of community-dwelling older adults in a typical region of China and explore the relationship between these dietary patterns and sarcopenia.

**Methods:**

We used data of the Nutrition and Health Follow-up Study of the Chinese Population in 2021. Food frequency questionnaires were used to obtain food items intake frequency during the last year. Appendicular skeletal muscle mass (ASM), muscle strength and physical performance were assessed according to the Asian Sarcopenia Working Group (AWGS2019) criteria. Exploratory factor analysis was used to identify dietary patterns. Logistic regression models were used to examine the association between dietary patterns and sarcopenia.

**Results:**

A total of 1,967 participants over the age of 65 were included in the study, and the prevalence of sarcopenia was 17.0%. According to the factor loadings of all of the 18 food groups, three dietary patterns were identified. These dietary patterns include the diversified dietary pattern, which is mainly characterized by the intake of soybeans, fungi and algae, animal meat, fruits, and legumes; the traditional dietary pattern, which is mainly defined by the consumption of rice, pork, poultry, vegetables, and aquatic products; and the wheat-based dietary pattern, which is mainly characterized by the intake of wheat, tubers, and other cereals. The diversified dietary pattern (OR = 0.54, *p* < 0.05) and the traditional dietary pattern (OR = 0.51, *p* < 0.05) were linked to a lower risk of developing sarcopenia, whereas the wheat-based dietary pattern (OR = 3.54, *p* < 0.05) was associated with a higher risk of sarcopenia. All three dietary patterns exhibited significantly correlated with muscle mass, grip strength, and physical performance (*p* < 0.05).

**Conclusion:**

Dietary patterns are associated with sarcopenia in community-dwelling older adults in China. Adopting a healthy and sensible balanced diet and avoiding a single dietary preference may reduce the risk of sarcopenia in older adults.

## 1 Introduction

Sarcopenia is defined as a decline in skeletal muscle mass and muscle strength or physical performance associated with aging (1). The global prevalence of sarcopenia among older adults remains significant, and it adversely impacts their quality of life, increasing the risk of falls (2), fractures (3), disability, incapacitation, and death (4). Research indicates that sarcopenia is also associated with a heightened risk of age-related diseases such as osteoporosis (5), cardiovascular disease (6), metabolic syndrome (7), and cognitive impairment (8). Nevertheless, the pathogenesis of sarcopenia in older adults remains poorly understood. Like many age-related diseases, sarcopenia is influenced by a variety of factors, including genetics, environment, nutritional status, and physical activity. An emerging body of research underscores the critical role of dietary nutritional factors in the onset and progression of muscle wasting, highlighting their potential to mitigate this condition. The intake of nutrients such as protein (9), vitamin D (10), and omega-3 polyunsaturated fatty acids (11) is effective in improving muscle health. However, achieving adequate nutrient intake in daily life is often best accomplished through diverse dietary combinations, as the complex interactions among foods may introduce biases in the observed associations between diet and disease.

Dietary pattern (DP) analysis has been identified as a more comprehensive approach to dietary characterization than a single food or nutrient analysis. Dietary patterns serve as generalized representations of the types, amounts, and proportions of foods consumed in a diet. It is viewed holistically in terms of the complex interrelationships between various foods and nutrients, reflecting an individual’s actual dietary habits. The use of dietary patterns to analyze their association with health is to some extent more comprehensive and accurate. Studies have been conducted to analyze the relationship between diet and muscle health using dietary pattern analysis methods such as *a priori* and a posteriori methods. Among the more consistent findings are that the Mediterranean diet has a positive effect on muscle function (12, 13). A study conducted in Japan revealed that the dietary pattern with a high intake of fish, soy products, potatoes, most vegetables, mushrooms, seaweeds, and fruits and a low intake of rice was inversely associated with muscle wasting disorders among community-dwelling older adults (14). An analysis of data from a Chinese longevity cohort study showed that middle-aged and older adults with healthier dietary patterns had decreased odds of developing muscle-wasting disease (15). Furthermore, another study found that individuals with a balanced dietary pattern and a rice-meat dietary pattern had higher levels of skeletal muscle mass, strength, and function (16). However, it is important to note that most of these studies were conducted in specific regions, and the regionally characterized dietary patterns may have limited applicability when extrapolated to other regions or populations.

This study based on the Nutrition and Health Follow-up Study of the Chinese Population in 2021, cross-sectional analyses was used to investigate the association between dietary patterns and sarcopenia among community-dwelling older adults in China. Specifically, it investigated the dietary and muscular status of community-dwelling older adults in five Chinese provinces: Jiangsu, Fujian, Shanxi, Chongqing, and Guangxi. Dietary patterns were extracted by exploratory factor analysis based on the eigenvalues of food intake. The study aimed to explore the relationship between these dietary patterns and sarcopenia, identifying patterns that may offer potential protective effects against the condition.

## 2 Materials and methods

### 2.1 Participants and study design

**Figure 1:**
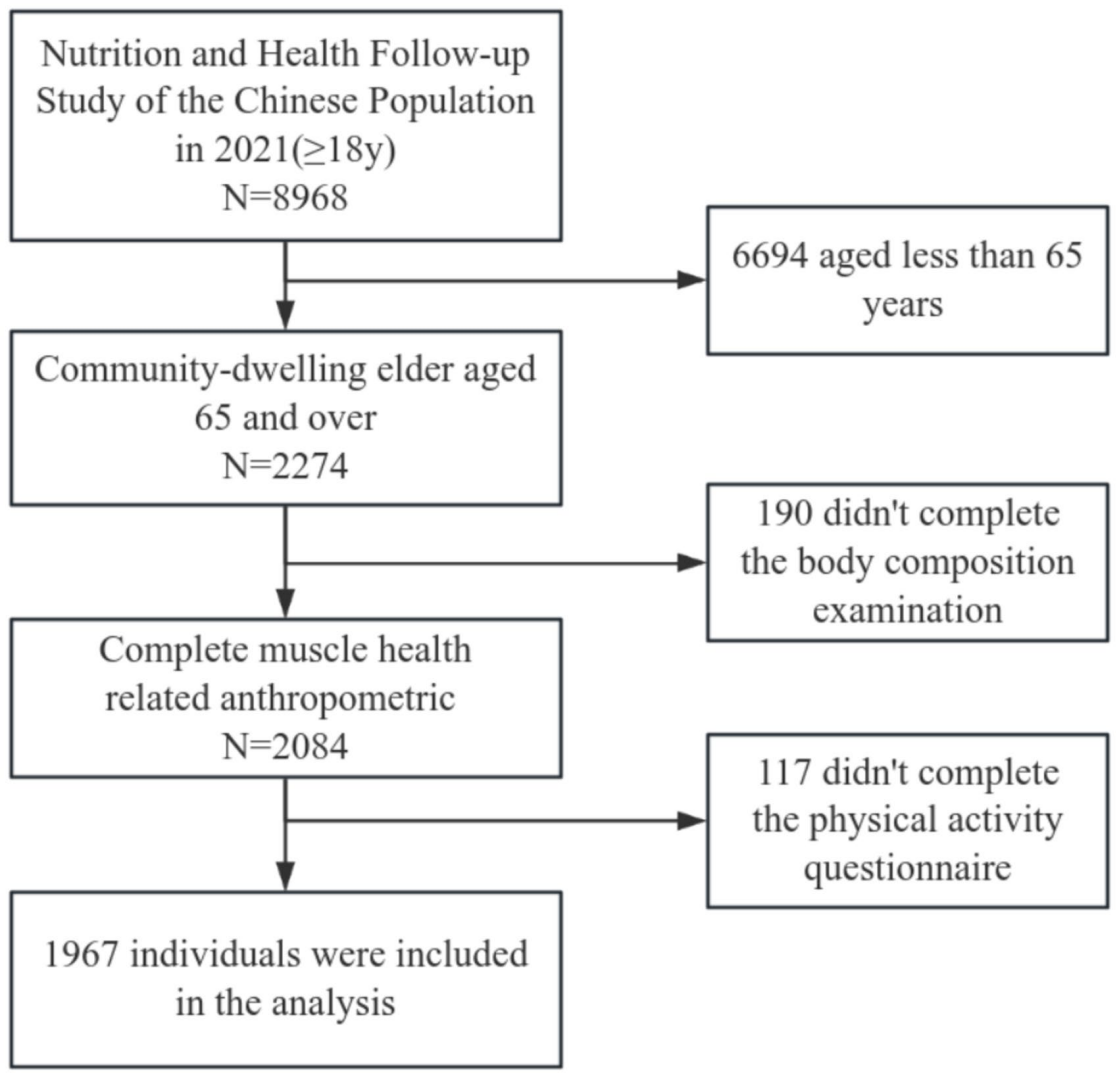
Flowchart of the participant selection process.

The data for this study came from the Nutrition and Health Follow-up Study of the Chinese Population in 2021. The study was based on the 2010–2012 National Population Nutrition and Health Surveillance. Based on 150 monitoring points in 31 provinces (autonomous regions and municipalities directly under the central government), and taking into account as much as possible the balanced distribution of stratification factors such as geography and urban/rural areas, the study chose Jiangsu and Fujian Provinces from the east, Shanxi Province from the center, and Chongqing Municipality and Guangxi Zhuang Autonomous Region from the west, making a total of five provinces for the follow-up survey. According to the pre-survey follow-up rate ranking, a systematic sampling method was used to determine 10 follow-up points. The follow-up survey was conducted on adult residents aged 18 years and above who had participated in the 2010–2012 China Population Nutrition and Health Status Monitoring in the selected follow-up sites. Therefore, cross-sectional analyses of the follow-up data of people aged 65 years and above were conducted in this study. Individuals were excluded if they were (i) under 65 years of age; (ii) limited in daily activities, disabled, or unable to complete basic physical and body composition measurements; (iii) suffering from a major physical or mental illness; or (iv) had communication barriers that prevented them from completing the questionnaire. All of the participants signed an informed consent before the survey. This study was approved by the Ethics Committee of National Institute for Nutrition and Health (NINH), Chinese Center for Disease Control and Prevention (CCDC), with the ethics number 2021–011. The study flowchart is shown in Figure 1.

### 2.2 Questionnaire and dietary intake assessment

Demographic information, living habits, and personal health status were obtained through the Basic Information Registry of Household Members and the Personal Health Questionnaire. The physical activity questionnaire includes physical activity and static behavior. All inquiries are conducted by questionnaires, and face-to-face inquiries are conducted by trained investigators in households. The verified version of a 75-item food frequency questionnaire (FFQ) was used in this study, food consumption frequency of these participants over a specified period was assessed based on the frequency of daily, weekly, monthly, or yearly intake. All survey protocols and questionnaires were validated and revised by the project team of the Institute of Nutrition and Health, Chinese Center for Disease Control and Prevention.

### 2.3 Dietary pattern assessment

Information on the 75 food items obtained from the FFQ survey was summarized and combined to obtain 18 food groups based on the food categories in the Chinese Food Composition Table (17). Detailed food groups categorization are shown in Supplementary Table S1. Exploratory factor analysis was used to extract dietary patterns based on 18 food groups. The common factors (i.e., dietary patterns) were retained based on eigenvalues greater than 1.0 and interpretability. Each pattern was named after the food group with the highest loadings. Factor scores for each pattern were calculated for all subjects by summing the intake of each food group and weighting them by their factor loadings. Each dietary pattern factor score was divided into four levels, Q1, Q2, Q3, and Q4, according to quartile spacing. Higher factor scores in a pattern indicate greater consistency in this pattern.

### 2.4 Anthropometric measures

Height was measured using a stadiometer (TZG type) and weight was measured using an electronic weighing scale (G&G TC-200 K). Body mass index was calculated as body weight (kg)/height (m)^2^. Waist circumference (WC), and calf circumference (CC) were measured twice by trained investigators, using a non-elastic tape. The average data of the two measurements of each circumference were used for the analysis. Grip strength was measured by an electronic grip strength meter (CAMRY, Model EH 101). Handgrip strength was measured twice for each hand in the standing position, and the greater recorded value was considered the maximal grip strength. Body composition includes skeletal muscle mass (SMM), percentage of body fat mass (PBF) was measured by the bioresistive antibody method InBody H20B (InBody, Korea). Appendicular skeletal muscle mass (ASM) was calculated as 80 percent of SMM (18), and Skeletal muscle mass index (SMI) = ASM/height(m)^2^. In addition, older adults’ level of physical performance is judged by a separate 5-time chair stand test. This test requires subjects to complete five consecutive rises and sits in a chair with a backrest without arm support, and the investigators records whether the test was completed and how long it took to complete the test.

### 2.5 Diagnosis of sarcopenia

Diagnosis criteria of sarcopenia recommended by the Asian Working Group for Sarcopenia in 2019 (AWGS2019) (1) were used in the present study. Subjects with low muscle mass and low grip strength or low gait speed could be diagnosed as sarcopenia. According to the cut-off values of components of sarcopenia recommended by AWGS2019, low skeletal muscle mass: male with SMI < 7.0 kg/m^2^ or female with SMI < 5.7 kg/m^2^. Low grip strength was defined as a grip strength of less than 28.0 kg in males and less than 18.0 kg in females. The criteria of low physical performance were that the subject with 5-time chair stands test more than 12 s.

### 2.6 Quality control

The national-level project team will formulate quality control methods for the Chinese Population Nutrition and Health Follow-up Study (2021) and supervise its implementation. The national-level project team will conduct unified training and assessment for investigators. Investigators who pass the assessment can participate in the fieldwork. Each follow-up site uses the information collection and management platform established uniformly by the national-level project team, and adopts computers and tablets for data collection, entry and uploading. The national-level project team is responsible for data cleaning with the follow-up sites as a unit, and timely feedback and correction when problems are found.

### 2.7 Statistical analysis

Categorical data were presented as numbers and percentages, and comparisons between groups were performed by chi-square test. The partial correlation coefficients were calculated by Spearman’s rank correlation, and a correlation matrix was performed to reflect the interrelationships between all food groups and between food and body measures. Logistic regression was used to analyze the relationships between dietary patterns and sarcopenia. The lowest quartile was defined as a reference, odds ratios (ORs) and 95% confidence intervals (CIs) were calculated in different models. Model 1 was a univariate logistic regression model; model 2 was adjusted by age, gender and region; model 3 was adjusted by age, gender, region, body mass index (BMI), exercise activity, sleeping time, static time, and smoke status. All of the statistical analyses were performed by SAS 9.4 and R 4.4.1. The threshold of significance was *p* < 0.05.

## 3 Results

### 3.1 Characteristics of the participants in the study

A total of 1967 subjects were included in this study, and the characteristics of participants by sarcopenia categories are shown in Table 1. The study population had a mean age of 72.8 ± 5.7 years, with 51.5% of participants being female. The prevalence of sarcopenia was 17.0% according to the AWGS2019 diagnostic criteria. There were significant differences between subjects with and without sarcopenia in terms of gender, age group, region, and physical activity. The prevalence of sarcopenia was found to be higher in males compared to females, and more pronounced in rural areas than in urban settings. The prevalence of sarcopenia in males and females by age group is shown in Supplementary Table S2. Additionally, individuals engaging in sufficient weekly hours of moderate to high-intensity physical activity exhibited a lower prevalence of sarcopenia. Notably, the prevalence was significantly higher among smokers (32.3 percent) than non-smokers (14.5 percent). Furthermore, subjects diagnosed with sarcopenia demonstrated lower BMI levels compared to those without the condition (*p* < 0.01). There was no significant difference in terms of sedentary time as well as sleep time.

**Table 1:** Participants characteristics.

	All	Non-sarcopenia	Sarcopenia	*χ* ^2^	*p*
	1967 (100)	1,632 (83.0)	335 (17.0)		
Gender				154.37	<0.01
Male	954 (48.5)	688 (72.1)	266 (27.9)		
Female	1,013 (51.5)	944 (93.2)	69 (6.8)		
Age				51.29	<0.01
65 ~ 69	760 (38.6)	670 (88.3)	89 (11.7)		
70 ~ 79	970 (49.3)	799 (82.4)	171 (17.6)		
80~	237 (12.1)	162 (68.4)	75 (31.6)		
Area				31.18	<0.01
Urban	1,178 (59.9)	1,023 (86.8)	155 (13.2)		
Rural	789 (40.1)	609 (77.2)	180 (22.8)		
Smoking				54.85	<0.01
No	1,682 (85.5)	1,439 (85.5)	243 (14.5)		
Yes	285 (14.5)	193 (67.7)	92 (32.3)		
BMI				191.41	<0.01
Low	76 (3.9)	34 (44.7)	42 (55.3)		
Normal	843 (42.9)	628 (74.5)	215 (25.5)		
Overweight	733 (37.3)	671 (91.5)	62 (8.5)		
Obesity	315 (16.0)	299 (94.9)	16 (5.1)		
Central obesity				125.77	<0.01
Yes	893 (45.4)	834 (93.4)	59 (6.6)		
No	1,074 (54.6)	798 (74.3)	276 (25.7)		
Exercise activity				6.05	0.01
<150 min/week	960 (48.8)	776 (80.8)	184 (19.2)		
≥150 min/week	1,007 (51.2)	856 (85.0)	151 (15.0)		
Sedentary time				0.09	0.96
<3 h	853 (43.4)	710 (83.2)	143 (16.8)		
3 ~ 5 h	602 (30.6)	499 (82.9)	103 (17.1)		
>5 h	512 (26.0)	423 (82.6)	89 (17.4)		
Sleeping time				0.24	0.62
<7 h	519 (26.4)	427 (82.3)	92 (17.7)		
≥7 h	1,448 (73.6)	1,205 (83.2)	243 (16.8)		

Categorical data are shown as *n* (%); BMI, Body-mass index.

### 3.2 Associations between food groups and anthropometric index

**Figure 2:**
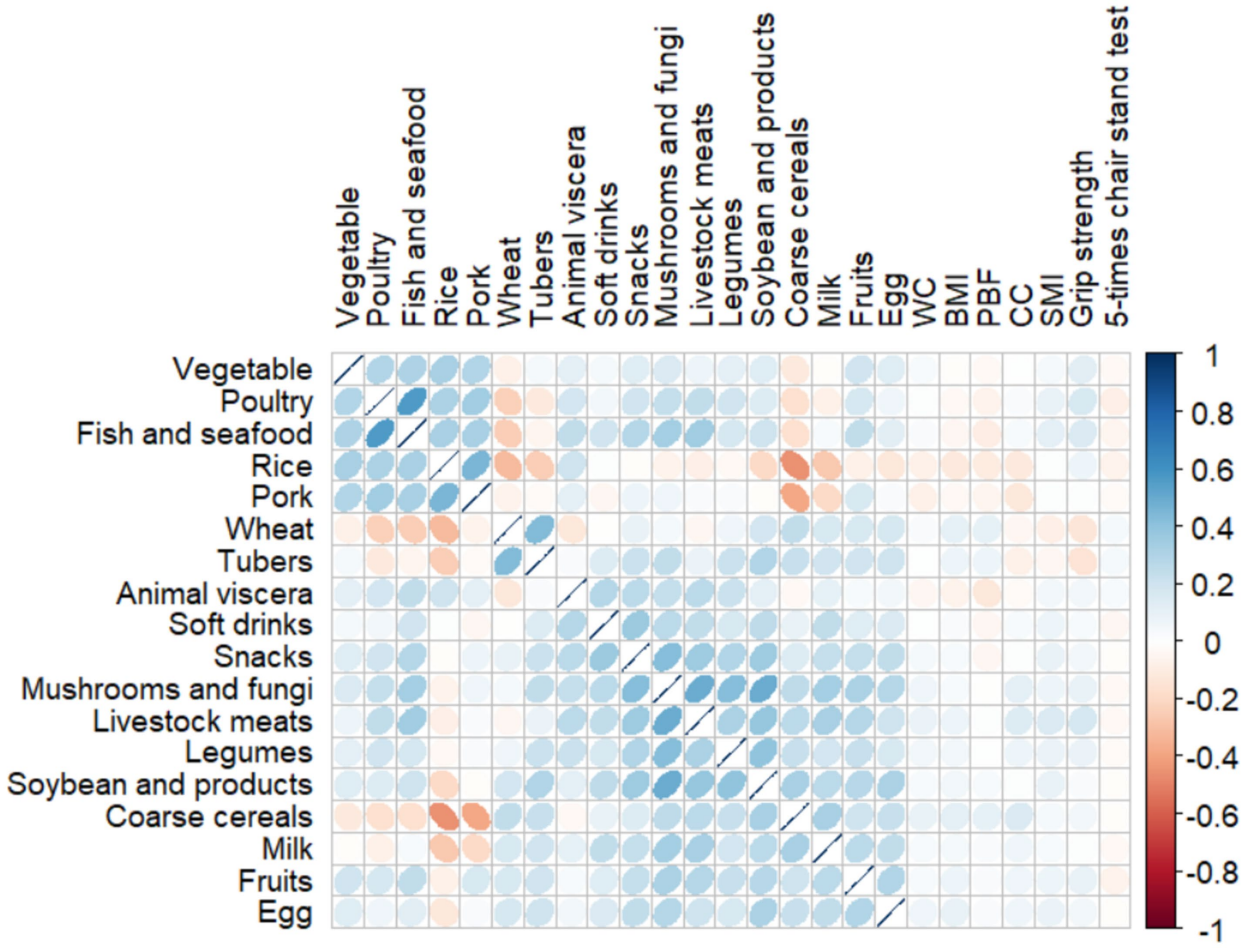
Consumption correlations among food groups and correlations between each food group and anthropometric characteristics.

Consumption correlations between all food groups and correlations between each food group and anthropometric characteristics are presented in a correlation matrix in Figure 2. After sorting and clustering the food items intake according to the correlation analysis by using Spearman rank correlation regression, intake of vegetables, poultry, aquatic products, rice and pork exhibited the strongest correlation, wheat and tubers also showed a strong correlation. Based on the food intake of this population, it is of practical significance to extract dietary patterns by exploratory factor analysis. In addition, some correlations were found between food groups and anthropometric indicators. Specifically, grip strength was negatively correlated with wheat and potatoes, and positively correlated with animal and poultry meat and aquatic products. However, the correlations between the food groups and sit-up time were weak.

### 3.3 Dietary patterns characteristic

**Figure 3:**
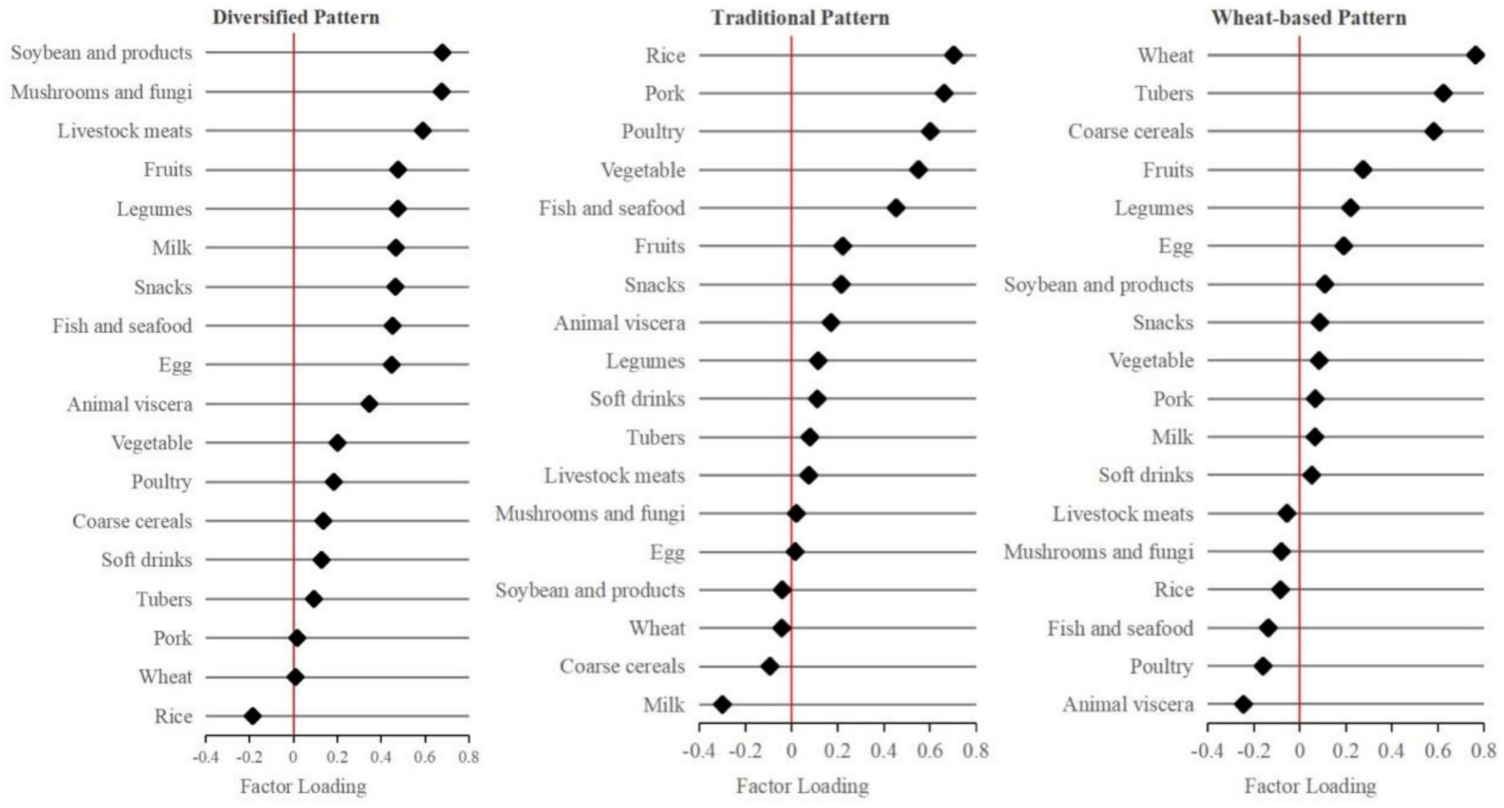
Factor loadings of different dietary patterns.

The KMO test statistic of 0.759 and Bartlett’s test of sphericity (*p* < 0.01) indicate a strong correlation among the food groups, making them suitable for factor analysis. Following the extraction of factors with eigenvalues greater than 1, and in conjunction with the scree plot and factor interpretability, three common factors were ultimately identified. The eigenvalues of these three common factors are 3.16, 1.98, and 1.38 respectively, and the explained variances are 17.6, 11.0, and 7.7%, respectively. These factors collectively accounted for more than 35% of the variance in food intake observed in this study. Based on the factor loadings of the 18 food groups, the identified factors corresponded to distinct dietary patterns, which were designated as the diversified dietary pattern, the traditional dietary pattern, and the wheat-based dietary pattern. The factor loadings of each food group for these three dietary patterns are shown in Figure 3.

### 3.4 Associations between dietary patterns and sarcopenia

The associations between dietary patterns and sarcopenia are presented in Table 2. Logistic regression analyses with the presence of sarcopenia as the dependent variable and adjusting for confounders showed that all three dietary patterns were significantly associated with sarcopenia. Specifically, the diversified dietary pattern (OR = 0.54, 95% CI = 0.35–0.82) and the traditional dietary pattern (OR = 0.51, 95% CI = 0.34–0.75) were linked to a lower risk of developing sarcopenia, whereas the wheat-based dietary pattern (OR = 3.54, 95% CI = 2.36–5.32) was associated with a higher risk of sarcopenia. In the highest quartile of the diversified dietary pattern and wheat-based patterns, the odds ratios (ORs) were elevated following adjustment for covariates. Conversely, for the traditional dietary pattern, the adjustment for covariates resulted in a reduction of the ORs for sarcopenia in both the crude and adjusted models. The results of exploring the relationship between sarcopenia and dietary patterns by gender are shown in Supplementary Table S3. After Modeling adjustments it was found that the diversified dietary pattern was a protective factor for the risk of developing sarcopenia in females, that the traditional dietary pattern was a protective factor for the risk of developing sarcopenia in males, and that the wheat-based dietary pattern was associated with sarcopenia in both males and females.

**Table 2:** Associations between Dietary patterns and sarcopenia.

Dietary pattern		Model 1		Model 2		Model 3	
		OR (95%CI)	*p*	OR (95%CI)	*p*	OR (95%CI)	*p*
Dietary pattern 1			<0.01		<0.01		0.03
	Q1	ref		ref		ref	
	Q2	0.81 (0.60, 1.10)	0.18	0.91 (0.65, 1.27)	0.57	0.93 (0.65, 1.33)	0.69
	Q3	0.57 (0.41, 0.79)	<0.01	0.71 (0.49, 1.01)	0.06	0.77 (0.52, 1.14)	0.19
	Q4	0.41 (0.29, 0.59)	<0.01	0.50 (0.34, 0.75)	<0.01	0.54 (0.35, 0.82)	<0.01
Dietary pattern 2			0.27		0.02		<0.01
	Q1	Ref		Ref		Ref	
	Q2	0.86 (0.62, 1.19)	0.37	0.97 (0.68, 1.38)	0.85	0.79 (0.54, 1.16)	0.22
	Q3	0.91 (0.66, 1.25)	0.55	0.92 (0.65, 1.31)	0.64	0.78 (0.54, 1.14)	0.20
	Q4	0.72 (0.51, 1.00)	0.05	0.60 (0.42, 0.86)	<0.01	0.51 (0.34, 0.75)	<0.01
Dietary pattern 3			<0.01		<0.01		<0.01
	Q1	Ref		Ref		Ref	
	Q2	1.35 (0.93, 1.96)	0.11	1.45 (0.98, 2.16)	0.06	1.79 (1.17, 2.74)	<0.01
	Q3	1.77 (1.23, 2.53)	<0.01	1.84 (1.26, 2.70)	<0.01	2.71 (1.79, 4.10)	<0.01
	Q4	2.37 (1.67, 3.36)	<0.01	2.27 (1.56, 3.28)	<0.01	3.54 (2.36, 5.32)	<0.01

Model 1: Crude model. Model 2: adjusted by age, gender, and region. Model 3: adjusted by age, gender, region, body mass index (BMI), exercise activity, sleeping time, sedentary time, and smoke status. Dietary pattern 1 score ranged from − 1.84 to 10.37; Q1 (−1.84 ~ −0.68), Q2 (−0.68 ~ −0.27), Q3 (−0.27 ~ 0.38), Q4 (0.38 ~ 10.37). Dietary pattern 2 score ranged from − 2.62 to 7.94; Q1 (−2.62 ~ −0.65), Q2 (−0.65 ~ −0.17), Q3 (−0.17 ~ 0.45), Q4 (0.45 ~ 7.94). Dietary pattern 3 score ranged from − 4.37 to 11.60; Q1 (−4.37 ~ −0.62), Q2 (−0.62 ~ −0.27), Q3 (−0.27 ~ 0.37), Q4 (0.37 ~ 11.60). Dietary Pattern 1—Diversified Pattern; Dietary Pattern 2—Traditional Pattern; Dietary Pattern 3—Wheat-based Pattern; BMI, Body mass index.

### 3.5 Association of dietary patterns with muscle mass, grip strength, and physical performance

**Figure 4:**
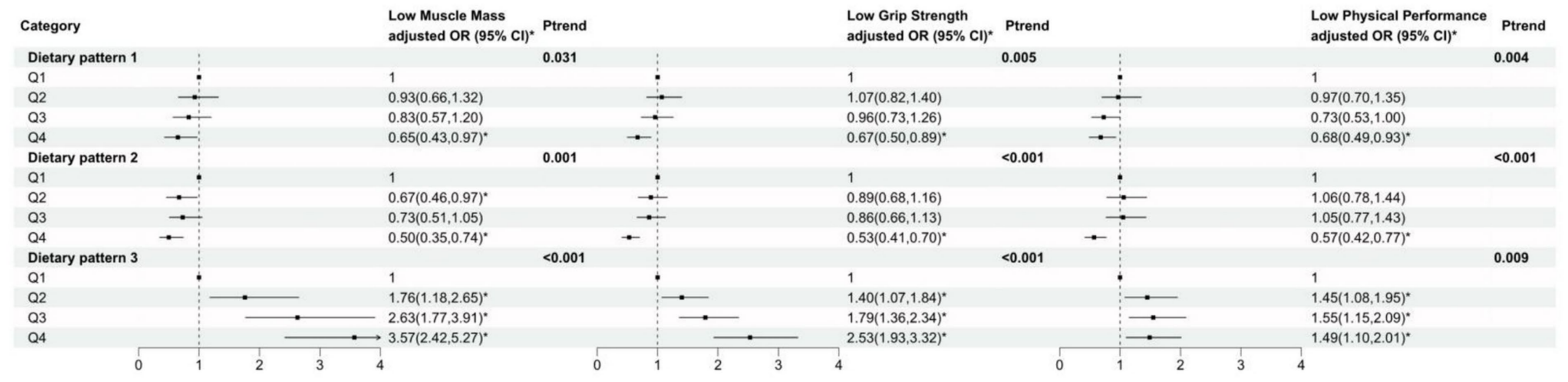
Dietary pattern correlated with muscle mass, grip strength, and physical performance. ^*^ *p* < 0.05.

Correlations between dietary patterns and anthropometric characteristics related to muscle health are shown in Figure 4. After adjusting for age, gender, region, body mass index (BMI), exercise activity, sleeping time, sedentary time, and smoke status, all three dietary patterns exhibited significantly correlated with muscle mass, grip strength, and physical performance. Specifically, higher quartiles of dietary pattern scores were negatively associated with low muscle mass, low grip strength, and low physical performance capacity compared to the lowest quartiles of dietary pattern 1 and dietary pattern 2. In contrast, Dietary Pattern 3 scores were strongly negatively correlated with the three muscle health indicators (*p*-trend is less than 0.05).

## 4 Discussion

In this study, we found that the prevalence of sarcopenia in this population was 17%, a result similar to the overall prevalence of sarcopenia in community-dwelling older adults over 65 years of age in China of 17.4% by Ren et al. (19). In this case, the prevalence of sarcopenia was higher in the older age group, and a Chinese epidemiological study reported that the prevalence of sarcopenia tended to increase with age in older adults (20). In addition, the results of the study showed that the prevalence was significantly higher in men than in women, and the same results were obtained in the study by Chen et al. (21). The main reason for this may be due to the difference in physiological factors such as hormones between males and females, as testosterone levels are often higher in males than females, and the decline in testosterone levels in males with and age may lead to a decrease in muscle mass (22, 23). In addition, the prevalence rate in rural areas is higher than that in urban areas, smokers are higher than non-smokers, and the differences in economic levels and individual lifestyles and diets in different regions may result in different nutritional status of the older adults, which may lead to differences in prevalence rates (19, 24). The prevalence of sarcopenia in the low-body-weight population in this study was 3.9%, which is consistent with other studies from Chinese communities (25). And the prevalence of sarcopenia was significantly higher in the low weight population than in the normal weight population. Physical nutritional status as reflected by body mass index may be one of the important reasons for the occurrence of sarcopenia (26, 27). In addition, we identified some dietary factors associated with sarcopenia and found that certain dietary patterns were associated with sarcopenia.

Dietary patterns represent groups of daily food consumption and can truly reflect daily dietary behaviors. In this cross-sectional study, we identified three primary dietary patterns among community-dwelling older adults individuals aged 65 years and older across five provinces in China. These dietary patterns include the diversified dietary pattern, which is mainly characterized by the intake of soybeans, fungi and algae, animal meat, fruits, and legumes; the traditional dietary pattern, which is mainly defined by the consumption of rice, pork, poultry, vegetables, and aquatic products; and the wheat-based dietary pattern, which is mainly characterized by the intake of wheat, potatoes, and other cereals. These dietary patterns are similar to the “grains-vegetables-pork” and “beans-mushroom” observed in the study of Shang et al. (28), although no dietary pattern with wheat whole grains as the main feature was observed. However, a survey conducted by Ding et al. (29) in rural China found that the dietary pattern of the older adults is mainly characterized by the intake of cereals. The dietary patterns identified from data on Chinese older adults individuals show many similarities, though some differences exist, which may be due to variations in dietary survey methods and the classification and naming of these patterns.

In exploring the relationship between three dietary patterns and sarcopenia, this study showed that the diversified dietary pattern and the traditional dietary pattern were both associated with a lower risk of sarcopenia after adjusting for age, sex, region, BMI, physical activity, sleep and sedentary time, and other confounding factors. A Japanese study indicated that a dietary pattern characterized by a high intake of fish, soy products, potatoes, most vegetables, mushrooms, seaweed, and fruits, along with a low intake of rice was associated with sarcopenia in community-dwelling Japanese older adults (14). This dietary pattern exhibited a negative correlation with sarcopenia and shared some similarities with the dietary pattern characteristics identified in this study. Meat, fish, eggs, milk, and soybean products are important sources of dietary protein. The diversified dietary pattern in this study contains a large amount of soybean food and livestock meat, both of which are rich in high-quality protein. Soybeans, mushrooms and algae foods are excellent sources of plant-based protein (30). Research shows that protein intake can promote muscle protein synthesis and help mitigate the age-related decline in muscle mass, strength, and functional capacity (31, 32). Therefore, high-protein dietary intake can reduce the risk of sarcopenia, which has also been confirmed in prospective studies (33, 34). In addition, soy isoflavones contained in soybeans and bioactive substances in bacteria and algae foods have been proven to have certain anti-inflammatory effects (35, 36). The consumption of anti-inflammatory foods can also enhance muscle health and reduce the risk of sarcopenia (37). Additionally, traditional dietary patterns that include food groups such as staple food groups, meat, and vegetables, and a relatively balanced dietary structure are protective against sarcopenia. A study also found that the adoption of a diversified diet in Chinese older adults may reduce the risk of sarcopenia in this population (38).

This study also found that after model adjustment, the wheat-based pattern was associated with a heightened risk of sarcopenia. However, a study conducted in Japan did not find an association between a dietary pattern characterized by wheat pasta and the risk of sarcopenia (39). This dietary pattern primarily consists of wheat, cereals, and potatoes, presenting a relatively simple nutritional structure that mainly provides the carbohydrates necessary for human health. The growth and maintenance of muscles require the support of proteins, fats, vitamins, and other essential nutrients (40–42). The intake of a single type of food makes it difficult to meet the comprehensive nutritional needs for muscle growth. In addition, this dietary pattern’s deficiency in animal foods may result in reduced high-quality protein intake, potentially leading to malnutrition, which can adversely affect muscle growth and repair, consequently contributing to sarcopenia (43). Findings indicate that single cereals intake is inadequate for muscle growth and may contribute to the risk of sarcopenia.

Further exploration of the relationships between the three dietary patterns and various anthropometric indicators related to muscle health found that all three dietary patterns were associated with muscle mass, handgrip strength, and physical performance after adjusting for other confounders. Notably, adherence to the diversified dietary pattern and the traditional dietary pattern is positively associated with appendicular skeletal muscle mass, handgrip strength, and physical performance. Research conducted by Elisa Mazza et al. (44) found a Mediterranean dietary pattern—characterized by increased consumption of beans, grains, fruits, vegetables, and limited intake of meat, fish, and eggs—that was also positively associated with both muscle mass and grip strength. Furthermore, the research also found that in older adults, greater dietary diversity may support the maintenance of physical functions such as grip strength and normal gait speed, although it did not appear to influence muscle mass (45). The wheat-based pattern score was negatively correlated with muscle mass, grip strength, and physical performance. However, there was several studies showed that a plant-based dietary pattern was more beneficial and effective than an animal-based diet for muscle mass in Chinese older adults (46). Plant-based foods in this study also included vegetables and soybeans etc. not limited to grains. As indicated above, a protein-rich, balanced and varied diet can improve muscle health and prevent muscle failure in older adults by increasing muscle mass, strength and physical performance.

Our study found that these dietary patterns simultaneously influence muscle health in terms of muscle mass, muscle strength, and physical performance. It suggests that higher adherence to the diversified dietary pattern and the traditional dietary pattern, which are dietary varieties, is associated with a lower prevalence of sarcopenia in older adults compared to low adherence to these dietary patterns. One of the strengths of this study is its approach to assessing dietary patterns, rather than focusing on single nutrients, which allows it to capture the complex relationship between diet and sarcopenia, leading to a more comprehensive understanding of the subject. In addition, the present study is a follow-up study based on a national monitoring survey, taking into account, as far as possible, the balanced distribution of stratification factors such as geography and urban/rural areas, and sampling the corresponding provinces. The survey data were obtained accurately, and the study sample is representative of typical regions in China. The study also provides epidemiological evidence for exploring the relationship between dietary patterns and sarcopenia in the Chinese population, due to the differences in dietary habits in different countries and regions.

This study also acknowledges several limitations. The conclusions are based on cross-sectional data, which precludes the establishment of causal relationships between dietary patterns and sarcopenia. More cohort studies are needed in the future to explore the relationship between diet and sarcopenia. In addition, the appendicular skeletal muscle mass used in the diagnosis of sarcopenia in this study was calculated based on the anatomy of the motor system, which indicates that the ASM is approximately 80% of the total skeletal muscle mass, so there may be certain measurement errors. Furthermore, this study used a food frequency questionnaire to investigate the frequency of various foods in the past year, but recall bias and other unidentified confounding factors cannot be avoided.

## 5 Conclusion

This study further suggests that dietary patterns are significantly associated with the prevalence of sarcopenia in older adults. The diversified dietary pattern and the traditional dietary pattern can reduce the risk of sarcopenia, whereas the wheat-based dietary pattern appears to increase the risk of sarcopenia. Therefore, the older adults should consume an appropriate amount of meat, soybeans, fungi, and algae foods, while also increasing the intake of whole grains, fruits, and vegetables. Adopting a healthy and reasonably balanced diet, and avoiding a single dietary preference is of great significance in preventing the occurrence of sarcopenia and promoting muscle health in older adults population, and it also provides a scientific basis for the development of dietary intervention guidance strategies for sarcopenia prevention and control.

## Data Availability

The datasets presented in this article are not readily available because the data presented in this study are not allowed to disclose. Requests to access the datasets should be directed to www.chinanutri.cn/.
