# Corrigendum: Association between dietary patterns and sarcopenia among community-dwelling older adults in five provinces of China: a cross-sectional study

**DOI:** 10.3389/fpubh.2025.1607874

**Published:** 2025-04-22

**Authors:** Rongchang Pu, Shanshan Jia, Xiaona Zhang, Qingqing Man, Dongmei Yu, Shuya Cai, Pengkun Song, Jian Zhang

**Affiliations:** ^1^Department of Geriatrics and Clinical Nutrition, National Institute for Nutrition and Health, Chinese Center for Diseases Control and Prevention, Beijing, China; ^2^Key Laboratory of Public Nutrition and Health, National Health Commission of the People's Republic of China, Beijing, China; ^3^Department of Epidemiology Nutrition, National Institute for Nutrition and Health, Chinese Center for Diseases Control and Prevention, Beijing, China

**Keywords:** sarcopenia, dietary patterns, older adults, muscle strength, muscle mass, muscle function

In the published article, there was an error in the Funding statement. The incorrect funder was listed.

The correct Funding statement appears below.

