# Supplementary material for: Association between dietary patterns and sarcopenia among community-dwelling older adults in five provinces of China: a cross-sectional study
Source: Front Public Health. 2025 Feb 28;13:1556033. doi: 10.3389/fpubh.2025.1556033 (PMC11906289; doi:10.3389/fpubh.2025.1556033)
Supplement: Supplementary file 1 [file Table_1.DOCX]

Table S1 Details of food group categorisation

| Food Group | Food species |
| --- | --- |
| Rice | Rice and its products |
| Wheat | Wheat flour and products, fried pasta products |
| Coarse cereals | Other cereals and products |
| Tubers | Tubers (potato/taro/sweet potato, etc.) |
| Soybean and products | Soybeans, soya milk, tofu, other soya products, etc. |
| Legumes | Legumes (green/red/flower beans, etc.) |
| Vegetable | Fresh and dried vegetables |
| Fruits | Fresh and dried fruits |
| Mushrooms and fungi | Edible fungi, mushrooms, kelp, etc. |
| Pork | Pork and its products |
| Livestock meats | Beef, lamb, etc. |
| Poultry | Poultry |
| Animal viscera | Animal viscera |
| Fish and seafood | Marine fish, freshwater fish, shrimp, crab, etc. |
| Egg | Eggs |
| Milk | Liquid milk, milk powder, yoghurt, cheese, etc. |
| Soft drinks | Sugary drinks, coffee, tea, etc. |
| Snacks | Bread, biscuits, chocolate, puffed food, etc. |

Table S2 The prevalence of sarcopenia in males and females by age group

|  | All | Nonsarcopenia | Sarcopenia | χ^2^ | *P* |
| --- | --- | --- | --- | --- | --- |
| Male |  |  |  | 43.43 | <0.01 |
| 60~69 | 352(36.9) | 284(80.7) | 68(19.3) |  |  |
| 70~79 | 486(50.9) | 347(71.4) | 139(28.6) |  |  |
| 80~ | 116(12.2) | 57(45.1) | 59(50.9) |  |  |
| Female |  |  |  | 9.65 | <0.01 |
| 60~69 | 408(40.3) | 387(94.9) | 21(5.2) |  |  |
| 70~79 | 484(47.8) | 452(93.4) | 32(6.6) |  |  |
| 80~ | 121(11.9) | 105(86.8) | 16(13.2) |  |  |

Table S3 Associations between Dietary patterns and sarcopenia in different gender.

| Dietary pattern |  | Male | | Female | |
| --- | --- | --- | --- | --- | --- |
|  |  | OR (95%CI) | *P* | OR (95%CI) | *P* |
| Dietary pattern 1 |  |  |  |  |  |
|  | Q1 | ref |  | ref |  |
|  | Q2 | 1.13(0.74,1.73) | 0.57 | 0.59(0.29,1.18) | 0.14 |
|  | Q3 | 0.75(0.47,1.19) | 0.23 | 0.85(0.42,1.74) | 0.67 |
|  | Q4 | 0.63(0.39,1.04) | 0.07 | 0.37(0.15,0.90) | **0.03** |
| Dietary pattern 2 |  |  |  |  |  |
|  | Q1 | Ref |  | Ref |  |
|  | Q2 | 0.61(0.38,0.98) | 0.04 | 1.45(0.70,3.00) | 0.32 |
|  | Q3 | 0.66(0.42,1.04) | 0.07 | 1.33(0.64,2.78) | 0.45 |
|  | Q4 | 0.43(0.27,0.68) | **<0.01** | 0.79(0.33,1.90) | 0.60 |
| Dietary pattern 3 |  |  |  |  |  |
|  | Q1 | Ref |  | Ref |  |
|  | Q2 | 1.69(1.03,2.78) | 0.04 | 2.01(0.87,4.65) | 0.10 |
|  | Q3 | 2.38(1.46,3.85) | **<0.01** | 3.84(1.70,8.67) | **<0.01** |
|  | Q4 | 3.73(2.32,5.99) | **<0.01** | 2.76(1.12,6.47) | **0.02** |

The logistic regression model adjusted by age, region, body mass index (BMI), exercise activity, sleeping time, sedentary time, and smoke status.
